# Malate metabolism mediated by the cytoplasmic malate dehydrogenase gene *MdcyMDH* affects sucrose synthesis in apple fruit

**DOI:** 10.1093/hr/uhac194

**Published:** 2022-11-01

**Authors:** Lihua Zhang, Changzhi Wang, Runpu Jia, Nanxiang Yang, Ling Jin, Lingcheng Zhu, Baiquan Ma, Yu-xin Yao, Fengwang Ma, Mingjun Li

**Affiliations:** State Key Laboratory of Crop Stress Biology for Arid Areas/Shaanxi Key Laboratory of Apple, College of Horticulture, Northwest A&F University, Yangling 712100, Shaanxi, China; The Key Laboratory of Biology and Genetic Improvement of Horticultural Crops (Northeast Region), Ministry of Agriculture and Rural Affairs, College of Horticulture & Landscape Architecture, Northeast Agricultural University, Harbin 150030, China; State Key Laboratory of Crop Stress Biology for Arid Areas/Shaanxi Key Laboratory of Apple, College of Horticulture, Northwest A&F University, Yangling 712100, Shaanxi, China; College of Horticulture Science and Engineering, Shandong Agricultural University, Tai’an, 271018, China; State Key Laboratory of Crop Stress Biology for Arid Areas/Shaanxi Key Laboratory of Apple, College of Horticulture, Northwest A&F University, Yangling 712100, Shaanxi, China; State Key Laboratory of Crop Stress Biology for Arid Areas/Shaanxi Key Laboratory of Apple, College of Horticulture, Northwest A&F University, Yangling 712100, Shaanxi, China; State Key Laboratory of Crop Stress Biology for Arid Areas/Shaanxi Key Laboratory of Apple, College of Horticulture, Northwest A&F University, Yangling 712100, Shaanxi, China; State Key Laboratory of Crop Stress Biology for Arid Areas/Shaanxi Key Laboratory of Apple, College of Horticulture, Northwest A&F University, Yangling 712100, Shaanxi, China; College of Horticulture Science and Engineering, Shandong Agricultural University, Tai’an, 271018, China; State Key Laboratory of Crop Stress Biology for Arid Areas/Shaanxi Key Laboratory of Apple, College of Horticulture, Northwest A&F University, Yangling 712100, Shaanxi, China; State Key Laboratory of Crop Stress Biology for Arid Areas/Shaanxi Key Laboratory of Apple, College of Horticulture, Northwest A&F University, Yangling 712100, Shaanxi, China

## Abstract

The types and proportions of soluble sugar and organic acid in fruit significantly affect flavor quality. However, there are few reports on the crosstalk regulation between metabolism of organic acid and sugar in fruit. Here, we found that the overexpression of cytoplasmic malate dehydrogenase genes (*MdcyMDHs*) not only increased the malate content but also increased the sucrose concentration in transgenic apple calli and mature fruit. Enzyme activity assays indicated that the overexpression of *MdcyMDH1* and *MdcyMDH5* enhanced sucrose phosphate synthase (SPS) activity in transgenic materials. RNA-seq and expression analysis showed that the expression levels of *SPS* genes were up-regulated in *MdcyMDH1*-overexpressed apple fruit and *MdcyMDH5*-overexpressed apple calli. Further study showed that the inhibition of *MdSPSB2* or *MdSPSC2* expression in *MdcyMDH1* transgenic fruit could reduce or eliminate, respectively, the positive effect of *MdcyMDH1* on sucrose accumulation. Moreover, some starch cleavage-related genes (*MdBAM6.1/6.2*, *MdBMY8.1/8.2*, *MdISA1*) and the key gluconeogenesis-related phosphoenolpyruvate carboxykinase *MdPEPCK1* gene were significantly up-regulated in the transcriptome differentially expressed genes of mature fruit overexpressing *MdcyMDH1*. These results indicate that alteration of malate metabolism mediated by *MdcyMDH* might regulate the expression of *MdSPSs* and SPS activity via affecting starch metabolism or gluconeogenesis, and thus accelerate sucrose synthesis and accumulation in fruit.

## Introduction

The composition and ratio of soluble sugars and organic acids are essential factors determining the flavor and organoleptic quality of fruit, which is widely believed to be a crucial driver to boost consumption [1]. Physiological studies have revealed that during the ripening of fleshy fruit, the organic acid content decreases and the accumulation of soluble sugar increases [2–5], suggesting an interaction between sugar and organic acid metabolism in fruits. Although considerable progress has been made to understand the regulatory mechanism of sugar and malic acid metabolism and transport, our knowledge about the potential crosstalk between organic acid and sugar metabolism in fruit remains rather limited. Comprehending the regulation pathway between soluble sugar and organic acid metabolism is thus of vital significance for improving fruit quality and enhancing the production of high-quality fruit.

Organic acid levels are involved in maintaining pH and contribute to fruit sensorial quality [6]. Malate is a primary organic acid accumulated in the vacuole to form the acidity of the fruit cell [7]. Whether malate can massively accumulate in the fruit cell relies highly upon the activity of enzymes related to malic acid synthesis and transportation [6]. Cytoplasmic malate dehydrogenase (cyMDH) primarily catalyzes the conversion from oxaloacetic acid (OAA) to malic acid in pineapple (*Ananas comosus*) [8], alfalfa (*Medicago sativa*) [9], wheat (*Triticum aestivum*) [10], cordifolia (*Aptenia cordifolia)* [11] and apple (*Malus domestica*) [12]. Malate, as an indispensable precursor of many metabolic pathways in fruit cells, can be used as a respiratory substrate in the tricarboxylic acid (TCA) cycle in mitochondria [3], or be imported into the gluconeogenesis pathway and be converted into soluble sugars [13].

The metabolism and transportation of organic acids and soluble sugars are mostly independent from each other. However, some studies have shown an intersection between sugar and acid metabolism and regulation. Echeverria pointed out that changes in organic acid content affect sucrose synthesis and catabolism in fruit [14]. In apple, when more photoassimilates were converted to malate, the overall distribution of assimilates to soluble sugar and starch would be changed at late fruit ripening [4]. When the mitochondrial malate dehydrogenase gene *mMDH* was targeted by RNA interference in tomato fruit, malate content was increased and soluble sugar content was decreased through a change in the redox state of cells [15]. In strawberry, overexpression of *FaMYB44.2* decreased sucrose accumulation by inhibiting the expression of the sucrose phosphate synthase (SPS) gene *FaSPS* and decreased the malate content in fruit [16]. These studies above indicate that organic acids and sugars in fruit are not metabolically isolated from each other. This is helpful to improve the fruit quality and elucidate the potential mechanisms of the crosstalk between carbohydrates and organic acids in fruit.

Apple is one of the world’s best-selling and most popular fleshy fruits. Unlike citrus, tomato and some other fruits, approximately 85% of organic acids in apple fruits are malic acid, which is mainly present as malate, while the soluble sugars are mainly fructose, sucrose, and glucose [7]. In the fruit of 155 apple accessions, the malic acid content showed a negative correlation with glucose content, whereas glucose content negatively correlated with sucrose content [5]. Interestingly, overexpression of a cytoplasmic malate dehydrogenase *MdcyMDH1* in apple calli or tomato fruit increased not only malic acid content but also sucrose content [12]. A recent study showed that the transcription factor MdbHLH3 directly activated the transcriptional expression of *MdcyMDH1* to coordinate carbohydrate distribution and malate accumulation in apple [17]. These results suggest that cytoplasmic malate dehydrogenase might be a node connecting sugar and acid in apple, while knowledge is scarce about how malate contributes to sugar metabolism, accumulation, and related processes.

In present study, *MdcyMDH1* overexpressed transgenic ‘Gala’ apples were used to explore the mechanism of malate affecting fruit soluble sugar. It was found that *MdcyMDH1* accelerated sucrose anabolism in the cytoplasm by affecting both the expression of the apple sucrose phosphate synthase gene *MdSPSB2/C2* and the SPS activity, and thus increased the sucrose content in fruit. Our findings improve our insight into the crosstalk between malate and sucrose in apple and provide potential pathways for optimizing the sugar and acid ratio inside fruit, which could improve fruit flavor quality.

## Results

### Overexpression of *MdcyMDH1* altered sugar content of apple fruit

**Figure 1:**
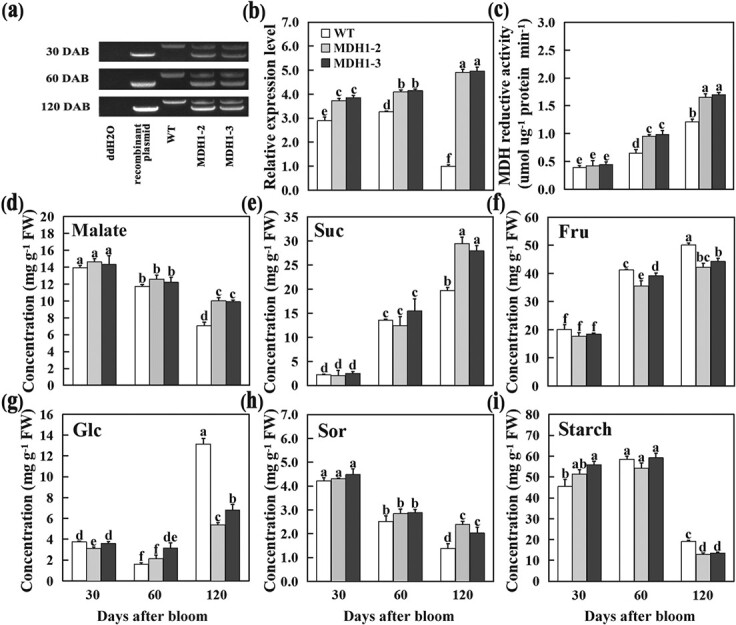
Functional analysis of *MdcyMDH1*. **a** Identification of *MdcyMDH1* overexpression in apple (MDH1–2 and MDH1–3) by PCR amplification. Cross-intron primer pairs were crafted to amplify coding sequences and genome sequences using WT and transgenic fruit DNA as templates. ddH2O and recombinant plasmid were used as a negative and positive control, respectively. **b** The mRNA relative expression levels of *MdcyMDH1* in wild-type (WT) and *MdcyMDH1*-overexpressed apple fruits at different stages of fruit development (30, 60, and 90 days after bloom). **c–i** MDH reductive activity, malate and carbohydrate levels in WT and *MdcyMDH1*-overexpressed apple fruits at different stages of fruit development. **c** MDH reductive activity. **d** Malate content. **e** Sucrose (Suc) content. **f** Fructose (Fru) content. **g** Glucose (Glc) content. **h** Sorbitol (Sor) content. **i** Starch content. The error bars show the standard deviation (SD) of data from three independent replicates. Different letters denote a significant difference (*P* < 0.05).

Two stable transgenic lines overexpressing *MdcyMDH1* driven by the CaMV35S promoter in the ‘Gala’ apple (MDH1–2, MDH1–3) were generated on the basis of previous experiments [12] (Fig. 1a), and no significant changes of per fruit weight between WT and two *MdcyMDH1-*overexpressed transgenic apples were observed (Fig. S1, see online supplementary material). To further explore how *MdcyMDH1* affects malate and sugar content in apple fruit, wild-type (WT) ‘Gala’ and transgenic apple fruits were harvested at different stages of fruit development (30, 60, and 90 days after bloom) for analysis of the *MdcyMDH1* expression level, MDH activity, and malate content.

In WT apple fruit, the expression of *MdcyMDH1* was high at the early stage of fruit development, and sharply decreased by 120 days after bloom (DAB), while in the fruits of the *MdcyMDH1-*overexpressed lines, the expression of *MdcyMDH1* remained high throughout fruit development (Fig. 1b). MDH reductive activity was significantly increased in the fruit overexpressing *MdcyMDH1* at 60 and 120 DAB compared with WT (Fig. 1c). In WT fruit, malate content continued to decrease with fruit development, while the overexpression of *MdcyMDH1* significantly increased the malate content only in mature fruit at 120 DAB, but had little effect on the malate content during early fruit development (Fig. 1d). In *MdcyMDH1-*overexpressed fruit, as malate content began to increase compared to WT, sucrose and sorbitol content were also increased significantly, while glucose and starch content were decreased at 120 DAB (Fig. 1e–i). These results suggest that *MdcyMDH1* functions to influence malate content and alter sugar content in apple fruit.

### *MdcyMDH1* overexpression changes activity of enzymes involved in sugar metabolism

**Figure 2:**
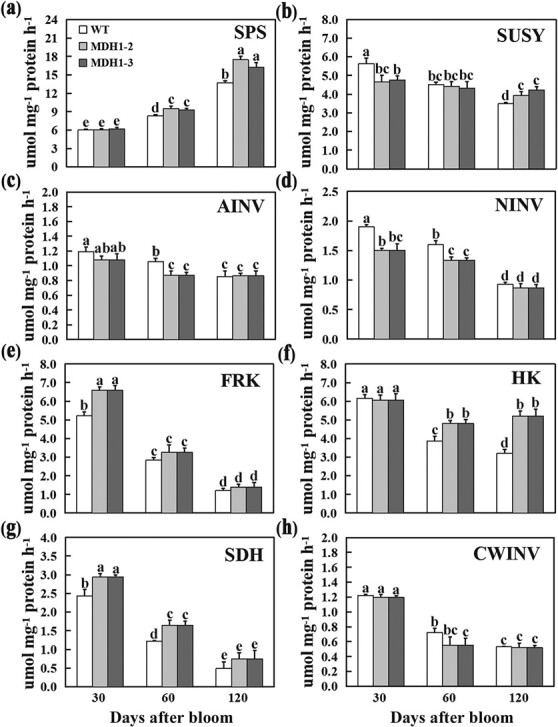
Measurement of enzyme activities related to sugar metabolism during fruit development in WT and *MdcyMDH1-*overexpressed apple fruit. **a** SPS, sucrose phosphate synthase; **b** SUSY, sucrose synthase; **c** AINV, vacuolar acid invertase; **d** NINV, neutral invertase; **e** FRK, fructokinase; **f** HK, hexokinase; **g** SDH, sorbitol dehydrogenase; **h** CWINV, cell wall invertase. Error bars show the standard deviation (SD) on the basis of three independent replicates. Different letters denote a significant difference (*P* < 0.05).

To explore the reasons for the altered sugar accumulation in *MdcyMDH1*-overexpressed apple fruit, the activities of enzymes involved in sugar metabolism were evaluated at various stages of fruit development. Sorbitol dehydrogenase (SDH) activity dropped continuously during fruit development and was significantly higher in *MdcyMDH1*-overexpressed fruit than in control (Fig. 2g). The activities of vacuolar acid invertase (AINV) and neutral invertase (NINV), both involved in sucrose breakdown, declined in *MdcyMDH1* transgenic fruit compared with control at 30 and 60 DAB, while the activity differences were not apparent between control and transgenic fruits at 120 DAB (Fig. 2c and d). Cell wall invertase (CWINV) activity decreased sharply from 30 to 60 DAB and then maintained relative stability to maturity in WT fruit, while decreasing slightly in transgenic fruit compared with the control only at 60 DAB (Fig. 2h). Sucrose synthase (SUSY), another crucial enzyme for sucrose decomposition in cell, showed a relatively stable activity in *MdcyMDH1-*overexpressed fruit but a sharp decrease in WT fruit over the fruit development period (Fig. 2b). The activity of SPS, associated with sucrose synthesis, continued to be elevated throughout fruit development and was significantly higher in *MdcyMDH1-*overexpressed fruit at 120 DAB than in control (Fig. 2a). The activity of hexokinase (HK) was significantly higher in *MdcyMDH1-*overexpressed fruit than in WT fruit at 60 and 120 DAB (Fig. 2f). Fructokinase (FRK) activity continued to decrease and was significantly higher in transgenic fruit than in control at 30 DAB (Fig. 2e).

### *MdSPSs* were up-regulated in *MdcyMDH1*-overexpressed apple fruit

To determine key genes during the process that up-regulated malate affected sugar content in *MdcyMDH1*-overexpressed apple, RNA-seq was conducted to profile gene expression in the WT and two *MdcyMDH1* transgenic apple fruits at 120 DAB. A total of 3815 differentially expressed genes (DEGs) (*P*-value <0.05 and |log2 (fold change)| ≥1) including 2508 up-regulated genes and 1307 down-regulated genes were found between control and two *MdcyMDH1* transgenic apple fruits (Fig. S2a, see online supplementary material). Among these DEGs, 24 genes were involved in major carbohydrate metabolism, and 19 genes were relevant to the tricarboxylic acid cycle (Fig. S2b, see online supplementary material).

**Figure 3:**
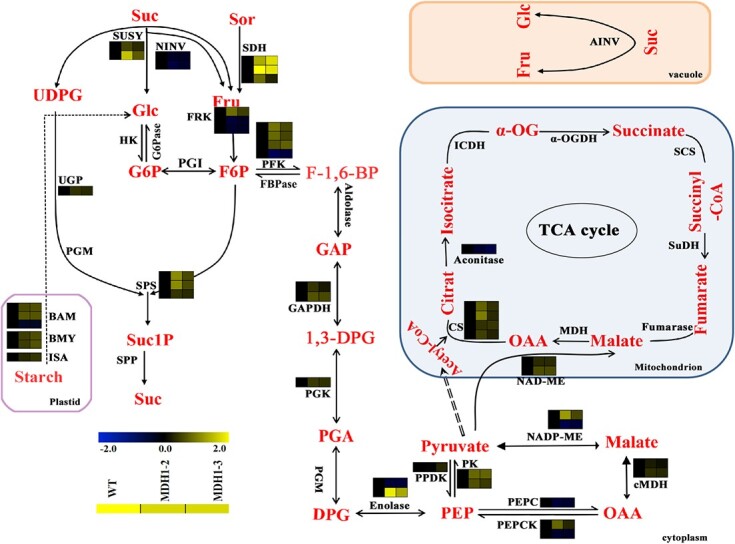
Effect of overexpression of *MdcyMDH1* on the expression of genes related to major sugar and acid metabolism in transgenic apple fruit. In each heatmap, the left-most box represents the expression level in WT ‘Gala’ fruits, set to 1. α-OGDH, α-oxoglutarate dehydrogenase; AINV, acid invertase; cMDH, cytoplasmic malate dehydrogenase; CS, citrate synthase; FBPase, fructose-1, 6-bisphosphatase; FRK, fructokinase; GAPDH, glyceraldehyde-3-phosphate dehydrogenase; HK, hexokinase; ICDH, isocitrate dehydrogenase; NAD-ME, NAD-malate enzyme; NADP-ME, NADP-malate enzyme; NINV, neutral invertase; PEPC, phosphoenolpyruvate carboxylase; PEPCK, phosphoenolpyruvate carboxykinase; PFK, phosphofructokinase; PGI, phosphoglucoisomerase; PGK, phosphoglycerate kinase; PGM, phosphoglucose mutase; PK, pyruvate kinase; PPDK, pyruvate phosphate dikinase; SCS, succinyl-coa synthase; SDH, sorbitol dehydrogenase; SPP, sucrose-phosphate phosphatase; SPS, sucrose phosphate synthase; SuDH, succinate dehydrogenase; SUSY, sucrose synthase; UGP, uridine diphosphate glucose pyrophosphorylase.

To verify the credibility of the RNA-seq expression profiling data, some important DEGs related to cytoplasmic malate metabolism were picked for expression analysis by quantitative real-time polymerase chain reaction (qRT-PCR). The expression levels of *MdcyMDH1, MdMDH2*, *MdPK1/2*, and *MdPEPCK1* were much higher in the two *MdcyMDH1* transgenic apple fruit (MDH1–2 and MDH1–3) compared to their levels in WT, while the expression levels of *MdPEPC* and *MdPEPCK2* in transgenic fruit were lower (Fig. S3, see online supplementary material; Fig. 1b). This qRT-PCR data was in agreement with the RNA-seq data (SI Appendix, Fig. S3, see online supplementary material). In addition, the overexpression of *MdcyMDH1* in the cytoplasm increased the expression of *MdNAD-ME* in mitochondria, leading to the up-regulation of *MdCS* gene expression in the tricarboxylic acid cycle pathway (Fig. 3). Taken together, these results indicate that the overexpression of *MdcyMDH1* accelerates malate metabolism in both cytoplasm and mitochondria.

For the DEGs involved in major carbohydrate metabolism, the expression of two sucrose synthase genes (*MdSUSYs*) increased, while two neutral invertases (*MdNINVs*) decreased. The expression of two of the three fructokinases (*MdFRKs*) were decreased (Fig. 3). It is worth noting that three key genes in sucrose synthesis, the *MdSPSs*, were significantly up-regulated in the transgenic lines (Fig. 3), which was consistent with the increased SPS enzyme activity in the *MdcyMDH1*-overexpressed apple fruit (Fig. 2a). This result may be an important reason for the increase of sucrose levels in mature *MdcyMDH1*-overexpressed apple fruit.

### Functional verification of *MdSPSs* in affecting sucrose content

The apple *MdSPS* family genes were obtained from the Apple Genome Database (https://www.rosaceae.org), and phylogenetic analysis was carried out with the four SPS proteins in *Arabidopsis thaliana* [18]. The MdSPS proteins were named based on their similarity to *Arabidopsis* SPS protein (Fig. S4, see online supplementary material). Subsequently, qRT-PCR was conducted to verify the expression levels of these three *MdSPS* genes in *MdcyMDH1* transgenic ‘Gala’ fruit. The results show that the expression levels of *MdSPSB2* and *MdSPSC2* in transgenic fruit increase by about four times as their levels in WT (Fig. S5, see online supplementary material).

**Figure 4:**
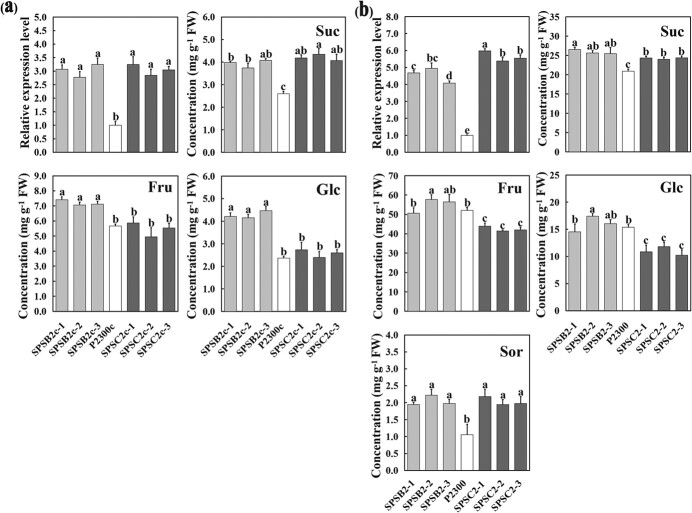
Carbohydrate levels in *MdSPSB2 and MdSPSC2* transgenic lines. **a** The mRNA relative expression level of *MdSPSB2/C2* and concentrations of sucrose, fructose, and glucose in control (P2300c) and *MdSPSB2-* and *MdSPSC2-*overexpressed apple calli (SPSB2c-1,2,3 and SPSC2c-1,2,3). **b** The mRNA relative expression level of *MdSPSB2/C2* and concentrations of sucrose, fructose, glucose, and sorbitol in control (P2300) and *MdSPSB2-* and *MdSPSC2-*overexpressed apple flesh (SPSB2–1,2,3 and SPSC2–1,2,3). The error bars show the standard deviation (SD) of three independent replicates. Different letters denote a significant difference (*P* < 0.05).

To confirm the function of *MdSPS* in altering sugar accumulation, the *MdSPSB2* and *MdSPSC2* overexpression vectors pCAMBIA2300-MdSPSB2 and pCAMBIA2300-MdSPSC2 were transformed into apple calli, obtaining *MdSPSB2-* and *MdSPSC2-*overexpressed calli lines. qRT-PCR analysis showed markedly elevated transcription levels of *MdSPSB2* and *MdSPSC2* in transgenic calli compared with the control (Fig. 4a). Overexpression of *MdSPSB2*/*MdSPSC2* significantly enhanced sucrose levels in transgenic calli, while a drastic decrease in glucose content was also observed in *MdSPSC2*-overexpressed calli (Fig. 4a).

To further characterize the effect of *MdSPSB2* and *MdSPSC2* on sugar levels in apple fruit, the overexpression vectors pCAMBiA2300-MdSPSB2/MdSPSC2 were transiently transformed into ‘Gala’ apple fruit via an *Agrobacterium*-mediated method. The overexpression of *MdSPSB2* or *MdSPSC2* elevated the accumulation of sucrose while the overexpression of *MdSPSC2* decreased the glucose content in ‘Gala’ apple flesh (Fig. 4b), which was similar with the trend of altered sugar content in apple calli (Fig. 4a). Additionally, increased sorbitol content was observed in *MdSPSB2*- and *MdSPSC2*-overexpressed apple flesh (Fig. 4b). These results indicate that *MdSPS* genes affect the changes of soluble sugars seen in apple calli and fruit.

### Sucrose concentration in the *sps* silencing of *MdcyMDH1*-transgenic apple

**Figure 5:**
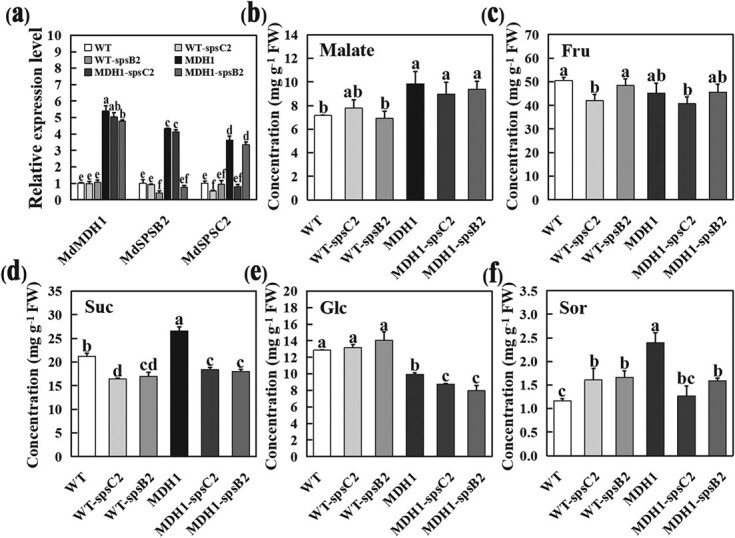
Effects of VIGS-mediated silencing of *MdSPSB2* and *MdSPSC2* on the soluble sugar content in WT and *MdcyMDH1*-overexpressed apple fruits. **a** The mRNA relative expression levels of *MdcyMDH1*, *MdSPSB2*, and *MdSPSC2* in WT and transgenic apple flesh (WT-spsB2, WT-spsC2, MDH1, MDH1-spsB2, MDH1-spsC2). **b–f** Malate and carbohydrate levels in WT and transgenic apple flesh. **b** Malate content. **c** Fructose content. **d** Sucrose content. **e** Glucose content. **f** Sorbitol content. The error bars show the standard deviation (SD) of three independent replicates. Different letters denote the significant difference (*P* < 0.05).

To genetically clarify if the effect of *MdcyMDH1* overexpression on the sucrose level is dependent on the high expression of *MdSPSB2* and *MdSPSC2*, *Agrobacterium* strain GV3101 carrying recombinant virus plasmids pTRV2-MdSPSB2 or pTRV2-MdSPSC2 were infiltrated individually into WT and *MdcyMDH1* transgenic mature ‘Gala’ fruits, with the empty vector pTRV2 as control, obtaining *MdSPSB2* and *MdSPSC*2 silenced lines in the background of WT and *MdcyMDH1*-overexpressed fruits (WT-spsB2/C2 and MDH1-spsB2/C2). In this study, silencing of *MdSPSB2/MdSPSC2* in both WT and *MdcyMDH1*-overexpressed fruits decreased the sucrose concentration (Fig. 5d).

Notably, the 50% ~ 60% reduction of the *MdSPSB2/MdSPSC2* transcripts in the WT fruits resulted in about a 20% reduction in sucrose content compared with that in the WT apple (Fig. 5a and d). Sucrose content in MDH1-spsB2/C2 apples decreased by 30% ~ 35% compared with *MdcyMDH1*-overexpressed fruit, while the difference in sucrose content between WT-spsB2/C2 and MDH1-spsB2/C2 transgenic fruit was not significant (Fig. 5d). Furthermore, the silencing of *MdSPSB2/MdSPSC2* did not cause significant changes in the malate contents in either the WT or *MdcyMDH1*-overexpressed fruits (Fig. 5b). These results indicate that upregulated *MdSPSB2/MdSPSC2* expression is the primary contributor altering the sucrose content in the mature apple fruit overexpressing *MdcyMDH1*.

### Accumulation of malate in cytoplasm increased the sucrose concentrations in apple fruit

**Figure 6:**
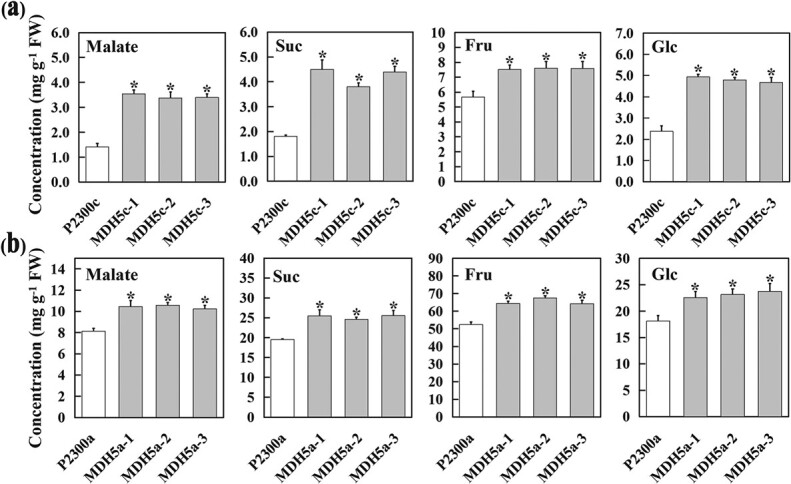
Malate and soluble sugar levels in *MdcyMDH5* transgenic lines. **a** The concentrations of malate, sucrose, fructose, and glucose in control (P2300c) and *MdcyMDH5-*overexpressed apple calli (MDH5c-1,2,3). **b** The concentrations of malate, sucrose, fructose, and glucose in control (P2300a) and *MdcyMDH5-*overexpressed apple fruit (MDH5a-1,2,3). The error bars show the standard deviation (SD) of three independent replicates. Asterisks indicate significant difference at *P* < 0.05.

Previous studies have shown that *MdWRKY126* enhances malate accumulation in apple calli and fruits by directly activating the expression of the cytoplasmic malate dehydrogenase gene *MdcyMDH5* [19]. In this study, the overexpression of *MdcyMDH5* resulted in the increased contents of sucrose, glucose, and fructose in transgenic apple calli and fruit (Fig. 6). These malate and sucrose content trends in *MdcyMDH5* transgenic lines were consistent with that in *MdcyMDH1*-overexpressed fruit. These results suggest that an increase of malate content in the cytoplasm can promote the accumulation of sucrose.

The enzyme activities and expression levels of eight genes in the SPS family were determined in *MdcyMDH5* transgenic calli. The SPS and SUSY activities in *MdcyMDH5* transgenic calli were significantly increased compared with the control, while the activities of CWINV and FRK decreased (Fig. S6, see online supplementary material). Meanwhile, the overexpression of *MdcyMDH5* significantly increased the transcript levels of *MdSPSA2.3* and *MdSPSB1* in transgenic calli (Fig. S7, see online supplementary material). These results further prove that the accumulation of malate in the cytoplasm increases sucrose content by up-regulating the expression of SPS genes.

## Discussion

The contents of soluble sugars and organic acids and their ratio largely affect the taste, quality, and commercial value of fresh apple [1]. Therefore, it is of great importance to explore the mutual regulatory mechanism of sugar and acid metabolism in apple fruit. Soluble sugars and organic acids in apple are not isolated from each other [4, 17]. Yao’s study showed that overexpression of cytoplasmic malate dehydrogenase *MdcyMDH1* in apple calli improved malate accumulation and also caused a change of sugar content [12], but the specific mechanism underlying this phenomenon was not pursued. To explore how acid metabolism affects sugar content, here, apple fruit stably overexpressing *MdcyMDH1* were used as material to investigate the effect of cytoplasmic malate dehydrogenase gene on sugar content.

MDH plays a vital role in seed germination [20], cell growth [21], embryo development [22], plant development and maturation [23], and stress resistance [24, 25]. Studies have showed that the MDH genes encoded a family of oxidoreductases localized to different organelles (cytoplasm, chloroplast, mitochondria, peroxisome) and use NAD^+^ or NADP^+^ as a coenzyme [9]. Cytoplasmic MDH (cyMDH) has been demonstrated to mainly catalyze the conversion from OAA to malate in most plants [8–10]. Overexpression of *MdcyMDH1* or *MdcyMDH5* increased malate accumulation in apple calli [12, 19]. Omics analysis indicated that mitochondrial MDH (mMDH) mainly catalyzed malate degradation during fruit ripening [3]. However, some studies have shown that mMDH could also catalyze the synthesis of malate in the presence of TCA reversible reaction conditions [26]. Overexpression of *MdmMDH12* in apple calli accelerated malate synthesis [27].

Most studies on the functions of acid metabolism genes have relied on transient transformation of fruit or stable expression of apple calli or a model plant due to the difficulty of stable transformation in apple and the 4–6 year juvenile phase for fruit set [17, 27]. In this study, the malate content in fruits grown on stable *MdcyMDH1-*overexpressed apple trees was determined. During fruit development, malate accumulated during the early stage and decreased by the late stage. This decrease of malate content may be caused by the increase of basal metabolism or the formation of sugars or secondary compounds during fruit ripening. The content of malate in mature *MdcyMDH1-*overexpressed fruit was significantly higher than that in WT, indicating the role of *MdcyMDH1* in malate accumulation of fruit. However, there was no significant difference in malate content at other periods, which may be because the expression level of *MdcyMDH1* in both transgenic and WT fruit was maintained at a high state before fruit ripening and *MdcyMDH1* expression level sharply decreased only in WT mature fruit. It is noteworthy that the overexpression of *MdcyMDH5* and *MdcyMDH1* all increased the accumulation of malate and sucrose in apple fruit, so we believed that the accumulation of malate might cause the change of sucrose content.

The content of soluble sugars in sink organs may be related to the accumulation of photosynthetic assimilates, but it was detected that the overexpression of *MdcyMDH1* did not significantly increase leaf photosynthesis [24], and the transgenic trees do not show difference in growth and development compared with wild type, which excluded the possibility to some extent that the overexpression of *MdcyMDH1* accelerated the accumulation of carbohydrates in source tissues. On the other hand, change of soluble sugar content in fruit vacuoles is highly regulated by sugar metabolism through various sugar synthesis and degradation genes [28, 29]. Transcriptomic differential expression analysis of *MdcyMDH1*-overexpressed apple revealed that the expression levels of *MdSPSB2* and *MdSPSC2*, which are homologous to the sucrose phosphate synthase genes *AtSPS3F* and *AtSPS4F* in *A. thaliana* [18], were significantly up-regulated (Fig. 3, Fig. S5, see online supplementary material). SPS is a key rate-limiting enzyme in the cytoplasmic sucrose synthesis pathway, catalyzing the synthesis of sucrose-6-phosphate from UDPG and F6P, followed by dephosphorylation to sucrose in the presence of sucrose-phosphate phosphatase (SPP) [30]. Studies showed that *SPS* gene expression was significantly up-regulated when sucrose content increased greatly during fruit development of bananas [31], melon [32], citrus [33], and sugarcane [34]. Similarly, in our study, SPS activity increased with fruit ripening. In mature apple fruit, the overexpression of *MdSPSB2* and *MdSPSC2* significantly accelerated sucrose accumulation (Fig. 4), which indicated that *MdSPSB2* and *MdSPSC2* were involved in the synthesis and accumulation of sucrose in apple. In addition, the silencing of *MdSPSB2* and *MdSPSC2* in the *MdcyMDH1*-overexpressed background of transgenic apple fruit significantly reduced sucrose level (Fig. 5). These results show that the overexpression of *MdcyMDH1* affects both the expression of *MdSPSB2* and *MdSPSC2* genes and the SPS activity, accelerating the accumulation of sucrose content in apple fruit.

The homeostasis of sugars and organic acids in the cytoplasm are regulated by multiple metabolism and transcription [6, 35]. Therefore, it is not unexpected that a large number of sugar- and acid-associated DEGs are present in the transcriptome of *MdcyMDH1*-overexpressed apple fruit (Fig. 3). In addition to *MdSPSB2* and *MdSPSC2*, several genes related to starch degradation (*BAM6.1/6.2*, *BMY8.1/8.2*, *ISA1*) were also up-regulated (Fig. 3), which might be another reason for the increased sucrose content in *MdcyMDH1* transgenic fruit. Studies have shown that SPS played an important role in carbon source allocation [36]. Higher SPS activity was associated with lower starch accumulation and higher sucrose content [37, 38]. Interestingly, the expression of the cytoplasmic localized phosphoenolpyruvate carboxykinase *MdPEPCK* was up-regulated (Fig. 3). Radiolabelling studies have clearly indicated that a portion of malate in ripe grape skins was converted to sugar via the gluconeogenic pathway, and PEPCK was considered the key enzyme converting malate to sugar [39]. In general, organic acid stored in ripe fruit provides only a small part of the substrate for fruit metabolism, while most of the carbon was sourced from sugar [40]. However, studies on peach and grape have shown that enhanced malate efflux from the vacuole induced gluconeogenesis, increasing the conversion from malic acid to sugar under certain circumstances [41, 42], which supported our result that the overexpression of *MdcyMDH1* greatly accelerated the accumulation of malate in the cytoplasm and induced the up-regulated expression of *MdPEPCK*, promoting the accumulation of sucrose.

Biochemical studies have shown that sugar homeostasis in cytoplasm is essential for basic cellular function, so the levels of various sugar components in the cytoplasm need to be strictly controlled within a certain range [35]. The synthesis of sucrose in pulp cells of *MdcyMDH1*-overexpressed apple consumed a large amount of glucose and fructose, but the reduction of fructose content in the fruit was slight. Unique to Rosaceae fruit, about 80% of fructose in apple fruit is produced by sorbitol degradation under the catalysis of SDH [43]. Among the DEGs in the *MdcyMDH1-*overexpressed fruit, we found three predicted *sorbitol dehydrogenase* genes that were significantly up-regulated (Fig. 3). These may accelerate sorbitol cleavage to balance fructose content in the cytoplasm in *MdcyMDH1* transgenic apple fruit. Because glucose, a direct carbon source, enters the glycolysis pathway to provide energy and serves as an intermediate product for cell metabolism, the glucose content in mature *MdcyMDH1-*overexpressed fruit was significantly reduced.

In summary, our study suggests that the cytoplasmic malate dehydrogenase gene *MdcyMDH1* acidifies apple fruit by accelerating the conversion of OAA to malate in cytoplasm. Meanwhile, *MdcyMDH1* overexpression upregulated the expression of the sucrose phosphosynthase genes *MdSPSB2* and *MdSPSC2*, leading to significantly enhanced accumulation of sucrose in apple fruit. This phenomenon was also observed in transgenic apple calli overexpressing the cytoplasmic *MdcyMDH5* gene, suggesting that the effect of cytoplasmic malate dehydrogenase on sucrose accumulation might be widespread. These findings are an initial exploration of the mechanism through which organic acid metabolism affects sugar content in apple fruit.

## Materials and methods

### Plant materials and growth conditions

‘Orin’ apple calli (*Malus × domestica*) were subcultured every 20 days on Murashige and Skoog (MS) solid medium supplemented with 1.0 mg/L 2,4-dichlorophenoxyacetic acid (2,4-D) and 1.0 mg/L 6-benzyla-minopurine (6-BA) at 23°C in dark conditions.

WT and *MdcyMDH1*-overexpressed ‘Gala’ apple trees [MDH1–2 (4 trees), MDH1–3 (3 trees)] were planted in 2013 in a garden belonging to Shandong Agricultural University, Tai’an (36°16’N, 117°6’E), Shandong, China at a spacing of 2.5 m × 1 m. The WT and transgenic trees are growing in a net room covered with 10-μm translucent nylon fabric to avoid genetic contamination for surrounding trees by pollen spread. The trees were trained as a spindle system. The crop-load was adjusted by hand-thinning to one fruit per 15 cm in crown to achieve a 10-mm king fruit size. During the growing season, fungicides and pesticides were sprayed at regular intervals throughout the growing season. The apple fruits were sampled in 2018. WT and *MdcyMDH1* transgenic ‘Gala’ apple fruit were collected at 30, 60, and 120 DAB for qRT-PCR, RNA-Seq and virus-induced vector injection assay. Each sample was sampled from six fruits with similar growth state on three trees and frozen immediately in liquid nitrogen and then stored at −80°C.

### Determination of organic acid, sugar, and starch content

Organic acid and sugar content were evaluated using GC–MS (gas chromatography–mass spectrometry) following the procedures as described by Li *et al.* [29]. Soluble sugars and organic acids were extracted with 1.4 mL of 75% (V/V) methanol for 0.1 g of sample, with 400 ppm ribitol was used as internal standard, by shaking at 900 rpm at 70°C for 30 min. The supernatants were separated and transferred into a mixture of 750 μL chloroform (CHCl_3_) and 1.4 mL ddH_2_O. After mixing and centrifugation, samples of 2 μL and 50 μL of the supernatant were dried and then derivatized with 40 μL methoxyamine hydrochloride and 60 μL N-methyl-N-trimethylsilyl-trifluoroace-tamide (MSTFA). Analysis of soluble sugar and organic acid contents was conducted on a GCMS-2010SE instrument (Shimadzu Corporation, Kyoto, Japan).

The determination of starch content was performed as described by Li *et al.* [28]. The remaining precipitate after the extraction with 75% methanol for the determination of sugar and acid was repeatedly cleaned with 80% (v/v) ethanol three times, mixed with 0.1 M KOH and boiled for 30 min to gelatinize the starch. α-amylase at pH 4.5 was added and incubated at 55°C for 1 h. Reducing sugar content was determined by 3, 5-dinitrosalicylic acid (DNS), and then converted to starch content.

### RNA-Seq and data assays

Total RNAs were extracted from WT and *MdcyMDH1*-overexpressed apple fruits at 120 DAB using RNAprep plant kit (Tiangen, Beijing, China). The RNA-Seq analysis was according to the methods as described by Zhu *et al.* [44].

### Phylogenetic analysis of the MdSPS family

*MdSPS* gene sequences were identified from *Malus × domestica* genome, GDDH13 v1.1 (https://www.rosaceae.org) by a protein–protein BLAST (BLASTp) analysis with the four *AtSPS* gene sequences reported in *A. thaliana* as templates. Phylogenetic analyses were performed with MEGA6.0 software (http://www.megasoftware.net/) via maximum likelihood method based on 1000 bootstrap replicates.

### Vector constructs and apple calli transformation

To generate the *MdSPSB2* and *MdSPSC2* transgenic apple calli, the complete CDS of *MdSPSB2* and *MdSPSC2* were cloned into the pCAMBIA2300 expression vector activated by CaMV35S promoter, forming the resulting vectors pCAMBIA2300-MdSPSB2 and pCAMBIA2300-MdSPSC2, which were then transformed into ‘Orin’ apple calli via an *Agrobacterium*-mediated method [45].

### RNA extraction and qRT-PCR reactions

Total RNA was extracted from apple calli and fruit flesh using RNAprep Plant Kit (Tiangen, Beijing, China) following the manufacturer’s instructions. Transcriptional expression of essential genes associated with sugar and acid metabolism was detected by quantitative real-time polymerase chain reaction (qRT-PCR) according to a previous report [46]. Each reaction was repeated three times to minimize error, and the transcript level was calculated via 2^-△△Ct^ method with *MdActin* (MDP0000752428) as internal reference. Primer sequences were designed through web software (http://biotools.nubic.northwestern.edu/OligoCalc.html), and their sequences are listed in Table S1, see online supplementary material.

### Determination of sugar and acid metabolism enzyme activities

MDH and sugar-related enzymes were extracted following the method of Wang *et al.* [24] and Li *et al.* [29], respectively. The whole process of enzyme extraction was performed at 4°C. Apple flesh (2 g) was extracted with 5 mL enzyme extraction buffer. The supernatant was treated by Sephadex G25 PD-10 desalination column (GE Healthcare, Buckinghamshire, UK) and collected for use.

For MDH reducing activity, the reaction system consisted of 50 mM Tris–HCl (pH 7.8), 2 mM MgCl_2_, 0.5 mM EDTA, 0.2 mM NADH, and 50 μL desalted extract. The reaction at 30°C was initiated after adding 2 mM oxaloacetate (OAA). The absorbance value at 340 nm was measured at 30 s intervals for 5 min by spectrophotometer.

For SDH activity, the reaction system consisted of 100 mM Tris–HCl (pH 9.6), 300 mM sorbitol, 1 mM NAD^+^ and 0.2 mL desalted extract. The absorbance value at 340 nm was measured by spectrophotometer after 3 min. The SDH activity was calculated according to the produced NADH amount.

For SPS activity, the reaction system consisted of 50 mM Hepes-KOH (pH 7.4), 1 mM EDTA, 4 mM MgCl_2_, 20 mM glucose 6-phsophate (G6P), 4 mM fructose 6-phosphate (F6P), 3 mM UDP-glucose (UDPG) and 250 μL desalted extract and was held at 27°C in a waterbath for 30 min, boiled for 3 min, then centrifuged. Supernatant (75 μL) was placed into a 1 mL mixture containing 50 mM Hepes-KOH (pH 7.0), 5 mM MgCl_2_, 0.3 mM NADH, 0.8 mM PEP, 14 U LDH, and 4 U PK. The absorbance value at 340 nm was measured by spectrophotometer.

For CWINV and AINV activity, the reaction system, consisting of 100 mM phosphate–citrate buffer (pH 4.8), 0.1 M sucrose and 125 μL desalted extract, was placed in a 37°C waterbath for 1 h, then 0.5 mL DNS was added and boiled for 5 min. The absorbance value at 540 nm was determined by spectrophotometer. The NINV activity was determined in a similar way to that of CWINV, except that the phosphate–citrate buffer was replaced by 100 mM Hepes-KOH (pH 7.2). Enzyme activity was calculated according to the produced glucose amount.

For SUSY activity, the reaction system consisted of 80 mM MES (pH 5.5), 5 mM UDP, 100 mM sucrose and 100 μL desalted extract was incubated at 27°C in a waterbath for 30 min. Then, the mixture was boiled for 5 min with 0.5 mL DNS. The absorbance value at 340 nm was measured by spectrophotometer.

For HK activity, the reaction system consisted of 50 mM Tris–HCl (pH 8.0), 2.5 mM ATP, 0.33 mM NAD^+^, 4 mM MgCl_2_, 1 U of G6PDH, 1 mM glucose, and 25 μL desalted extract was incubated at 30°C in a waterbath for 5 min. For FRK activity, the reaction system consisted of 50 mM Tris–HCl (pH 8.0), 2.5 mM ATP, 0.33 mM NAD^+^, 4 mM MgCl_2_, 1 U of G6PDH, 0.4 mM fructose, 1 U PGI and 60 μL desalted extract was incubated at 30°C in a waterbath for 5 min. The absorbance value at 340 nm was measured by spectrophotometer.

### Virus-induced silencing of genes in apple fruit

Specific antisense sequences of *MdSPSB2* (256 bp) and *MdSPSC2* (229 bp) were cloned and introduced into the tobacco rattle virus vector pTRV2, forming resulting vectors pTRV2-MdSPSB2 and pTRV2-MdSPSC2. These recombinant vectors were transformed into *Agrobacterium* strain GV3101 and individually injected into WT and *MdcyMDH1-*overexpressed apple fruit according to previous description [19]. The treated apple fruits were initially kept out of light for 24 hours to allow *Agrobacterium* penetration and then transferred to natural light for three days at room temperature. The area surrounding the fruit injection site was then sampled to analyse gene expression and the contents of malate and soluble sugar.

### Statistical analysis

Data analysis was carried out via one-way ANOVA or independent *t*-tests with significance level accepted at *P* < 0.05 using IBM SPSS Statistics 21. The values were presented as the mean ± standard deviation (SD) of biological triplicates.

## Acknowledgements

This work was supported by the Program for the Shaanxi science and technology innovation team project (2022TD-12), the National Natural Science Foundation of China (Grant Numbers 32072527) and the National Natural Science Foundation of Shaanxi Province (No. 2020JC-21). We thank the Horticulture Science Research Center at the College of Horticulture, NWAFU for their technical support in this work.

## Author contributions

M.L., F.M., Y.Y., and B.M. designed and supervised this research; L. Zha. and C.W. performed this research; L. Zhu, R.J., N.Y., and L.J. analysed data; L. Zha wrote the manuscript; M.L., F.M., Y.Y., B.M., L. Zha, and C.W. discussed this study and revised the manuscript.

## Data availability

The datasets generated and/or analysed during the current study are available from the corresponding author upon reasonable request.

## Conflict of interests

The authors declare no competing interests.

## Supplementary data

Supplementary data is available at *Horticulture Research* online.

## Supplementary Material

Web_Material_uhac194
