# Supplementary material for: Malate metabolism mediated by the cytoplasmic malate dehydrogenase gene MdcyMDH affects sucrose synthesis in apple fruit
Source: Hortic Res. 2022 Nov 1;9:uhac194. doi: 10.1093/hr/uhac194 (PMC9630971; doi:10.1093/hr/uhac194)
Supplement: Web_Material_uhac194 [file web_material_uhac194.pdf]

Supplemental Figures

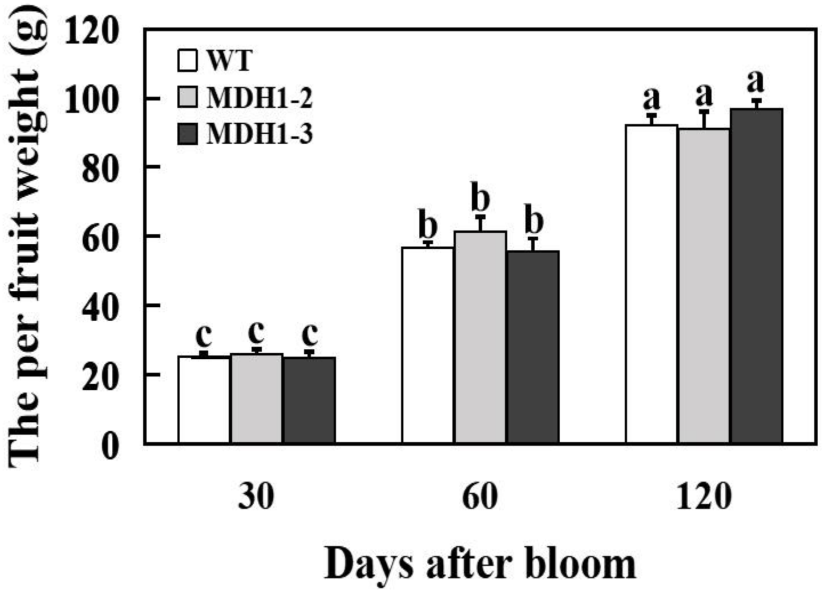

Fig. S1. The per fruit weight of WT and *MdcyMDH1*-overexpressed apple fruit (MDH1-2, MDH1-3) collected at 30, 60, and 120 days after bloom. The error bars show the standard deviation (SD) of three independent replicates. Different letters denote the significant difference ($P < 0.05$).

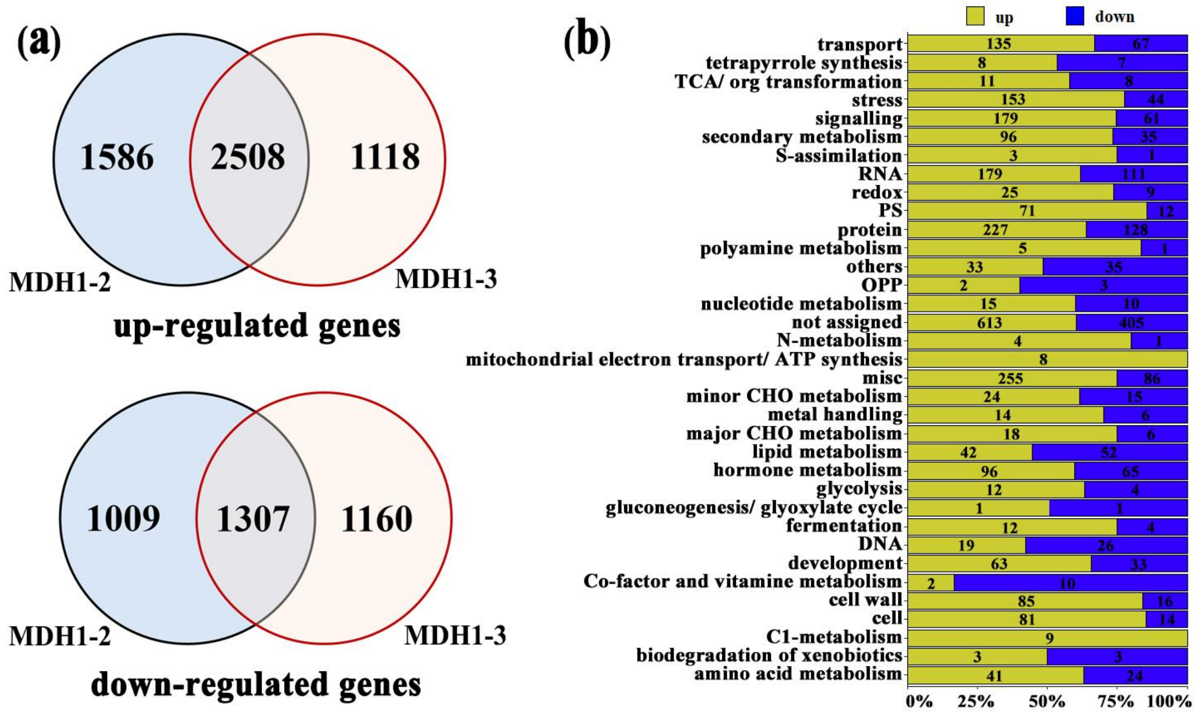

Fig. S2. Effects of overexpression of *MdcyMDH1* on gene expression related to malate and sugar metabolism in transgenic apple fruit. a Venn diagrams of the number of differentially expressed genes (DEGs) in the mature fruit of two *MdcyMDH1*-overexpressed lines (MDH1-2 and MDH1-3) compared with WT. **b** Functional category enrichment of DEGs. Yellow, significantly up-regulated genes; Blue, significantly down-regulated genes.

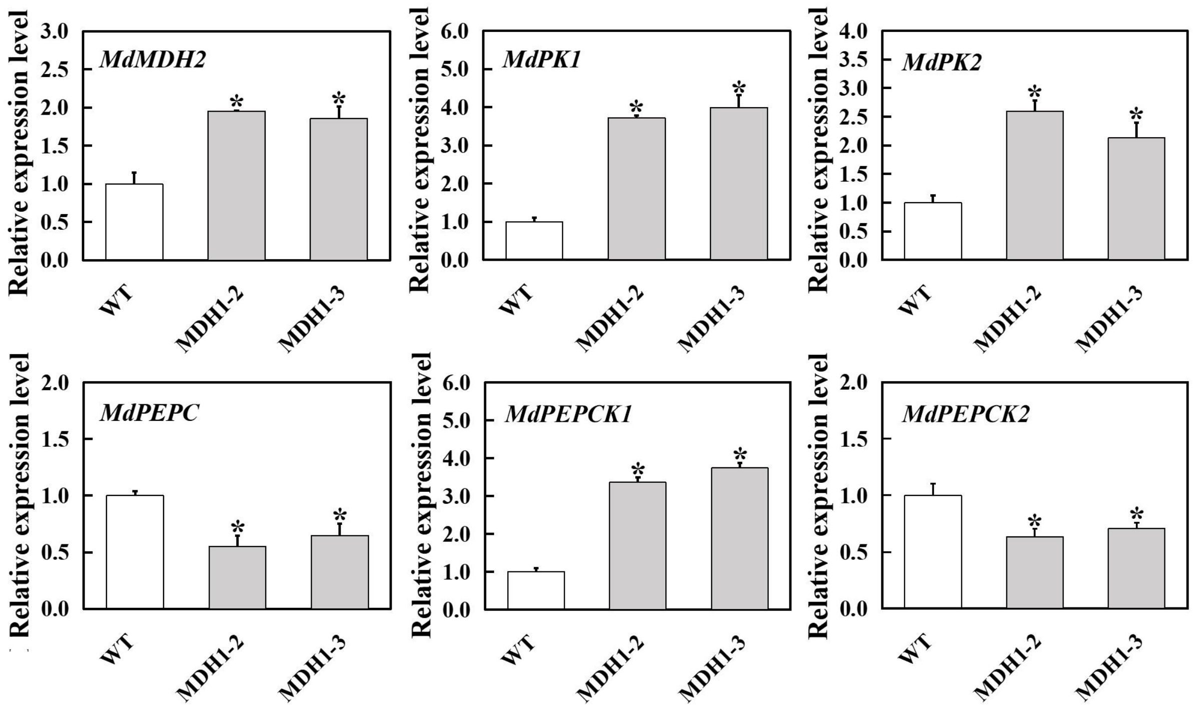

Fig. S3. Quantitative validation of transcript levels of key gene in the cytoplasmic malate metabolism pathway that were DEGs in *MdcyMDH1*-overexpressed apple fruit (MDH1-2, MDH1-3). The error bars show the standard deviation (SD) on the basis of three independent replicates. Asterisks indicate significant difference at $P < 0.05$.

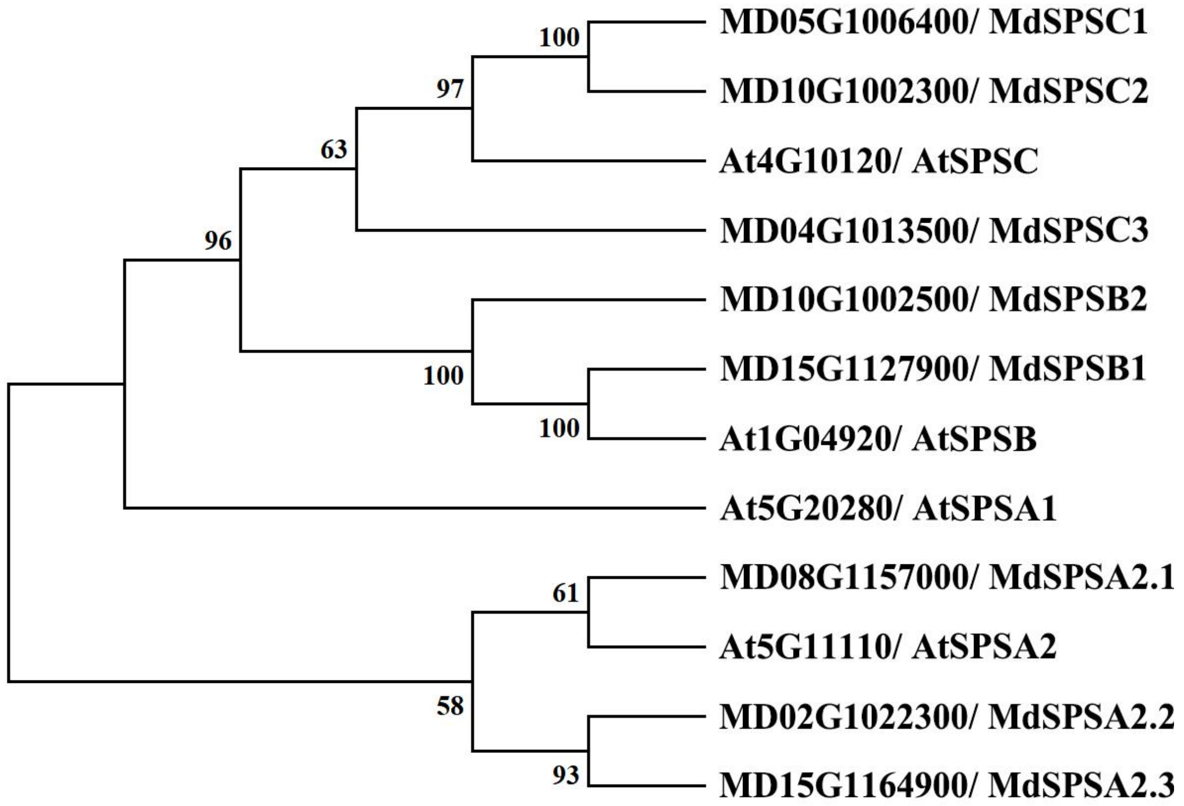

Fig. S4. Phylogenetic analysis of SPS. The SPS protein sequences from apple and arabidopsis were collected to construct a phylogenetic tree via the maximum likelihood method based on 1000 bootstrap replicates.

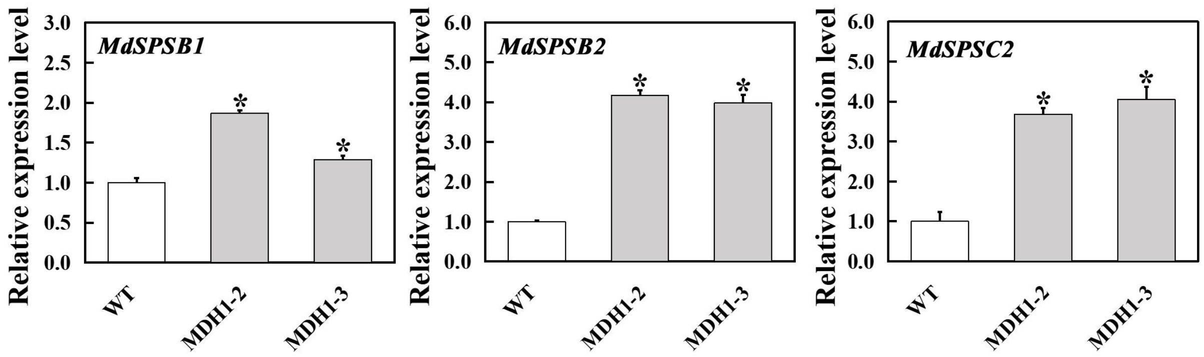

Fig. S5. Quantitative validation transcript levels of up-regulated *SPS* genes that were DEGs of *MdcyMDH1*-overexpressed apple fruit (MDH1-2, MDH1-3). The transcript levels of the *MdSPSs* were normalized to those of *MdActin*, and relative expression levels were calculated by the ddCT method. The error bars show the standard deviation (SD) on the basis of three independent replicates. Asterisks indicate significant difference at $P < 0.05$.

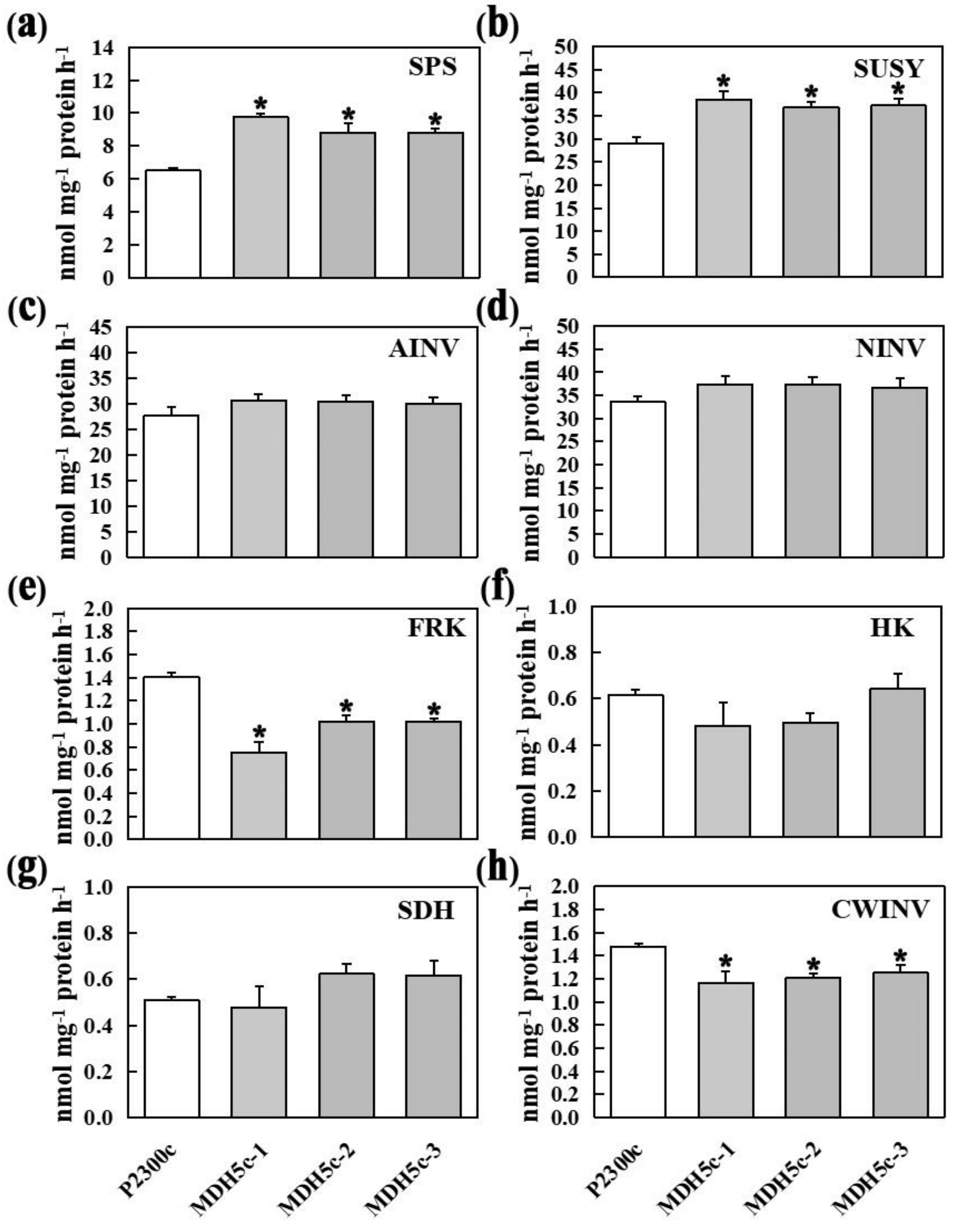

Fig. S6. Measurement of enzyme activity related to the sugar metabolism in control (P2300c) and *MdcyMDH5*-overexpressed apple calli (MDH5c-1,2,3). **a** SPS, sucrose phosphate synthase; **b** SUSY, sucrose synthase; **c** AINV, vacuolar acid invertase; **d** NINV, neutral invertase; **e** FRK, fructokinase; **f** HK, hexokinase; **g**. SDH, sorbitol dehydrogenase; **h** CWINV, cell wall invertase. The error bars show the standard deviation (SD) on the basis of three independent replicates. Asterisks indicate significant difference at $P < 0.05$.

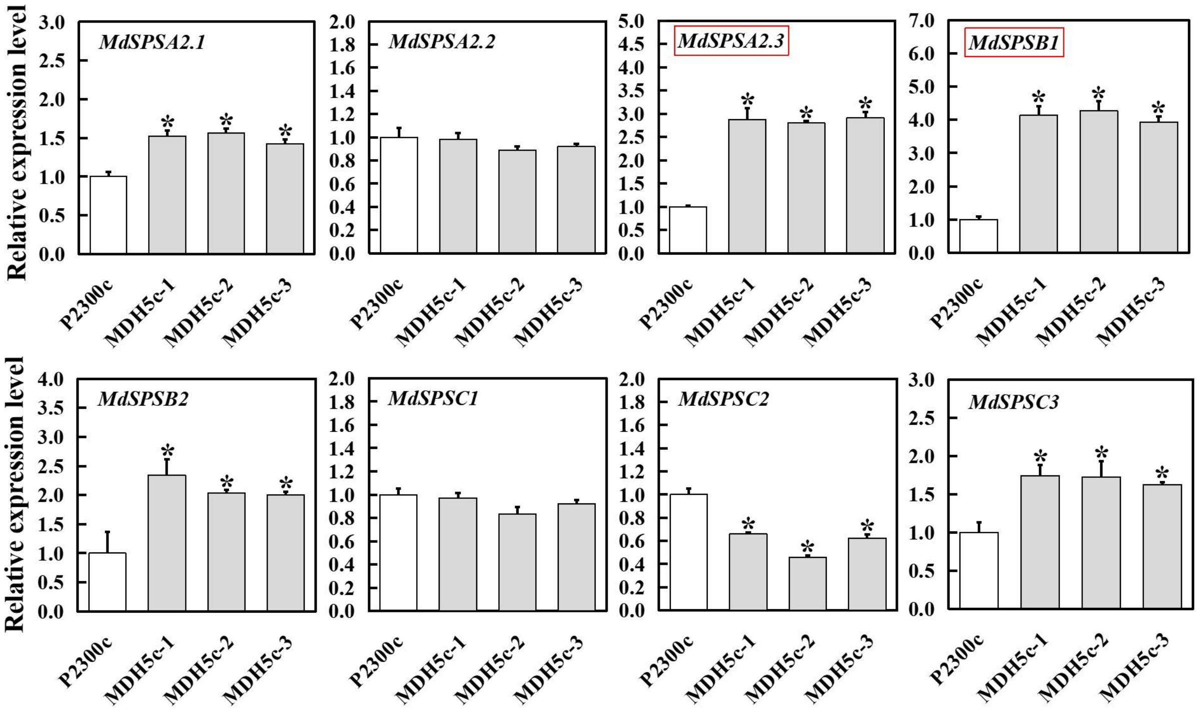

Fig. S7. Expression levels of *MdSPS* genes in control (P2300c) and *MdcyMDH5*-overexpressed apple calli (MDH5c-1,2,3). The transcript levels of genes were normalized to those of *MdActin*, and gene relative expression levels were calculated by the ddCT method. The error bars show the standard deviation (SD) on the basis of three independent replicates. Asterisks indicate significant difference at $P < 0.05$.

Table S1. The primer sequences designed in this study.

Primer name	primer sequence (5'-3')	Purpose
MdcyMDH1 (MD13G1214000)-F	CCA GTG ATT CTG CAC TTG CTC G	gene expression analysis
MdcyMDH1 (MD13G1214000)-R	TGG GAC TTG TAG ATG GAA ACG TTC TT	
MdSPSC2 (MD10G1002300)-F	CCC AGC TCT CGT GGA ACC T	
MdSPSC2 (MD10G1002300)-R	GTG GAT GTT CTT TAG GCC GTT CT	
MdSPSB2 (MD10G1002500)-F	CTC GCC CGT AAA AAG AAG CAG AT	
MdSPSB2 (MD10G1002500)-R	CAT GCA CGC TGA TTA GGA CAA TG	
MdSPSB1 (MD15G1127900)-F	CGG ATT TGG AAT TTG GCT CGC AA	
MdSPSB1 (MD15G1127900)-R	CAG TTC TCC ACT GCA TCA GTA GA	
MdSPSA2.2 (MD02G1022300)-F	GAG AGG AAC ACC AGA TTG GAG AAT T	
MdSPSA2.2 (MD02G1022300)-R	AATTCTAGGCAGTCTTCCTCTGTGA	
MdSPSA2.3 (MD15G1164900)-F	GAG TCC ACA GGA GAG GAA CAC	
MdSPSA2.3 (MD15G1164900)-R	CGG AGT TAA TTC TAG GCA GTT TGC T	
MdSPSA2.1 (MD08G1157000)-F	CGG AGG AGA GAA ACA CCA GGT	
MdSPSA2.1 (MD08G1157000)-R	TCT CCA TCG CAT CAG TGG AAT TGA T	
MdSPSC3 (MD04G1013500)-F	GGA GAA TCT GAG AGA TAG AGG ACA T	
MdSPSC3 (MD04G1013500)-R	CTT CGG ACA AGT CCT CCG ACA	
MdSPSC2 (MD10G1002300)-F	GTT TAC CAG TGG TCG CCA CCA	
MdSPSC2 (MD10G1002300)-R	TGG GGT GAC GGT TTC GGG AAT	
MdSPSC1 (MD05G1006400)-F	GAT GGC ATG GGA TGA CGC TCA	
MdSPSC1 (MD05G1006400)-R	CACGCACCAACCCATGCATAC	
MdMDH2 (MD16G1219000)-F	CGG TGA TTC TGC ACC TGC TTG	
MdMDH2 (MD16G1219000)-R	CCT TGC AGT TAG GAG CTG CAT G	
PEPCK1 (MD16G1217500)-F	CCT CTT GAC GGA GCC CAA GAT	
PEPCK1 (MD16G1217500)-R	GAAGAGGATGTTGTCCGGCTTG	
MdPEPC (MD09G1237900)-F	TTG GAT CCC GGG GAC TCC ATT	
MdPEPC (MD09G1237900)-R	TGAGTAGGATGTGCAGTCAGAACTA	
MdPK2 (MD07G1072900)-F	AAG AGA CAT TGG GAA ACC TGA AGG T	
MdPK2 (MD07G1072900)-R	ACA GAA GTA GTT TCA CTC CCC GTA A	
MdPK1 (MD02G1244000)-F	ACC TGA AGG TTG CAA TCA AGA GTA C	
MdPK1 (MD02G1244000)-R	ACT TCC CGT AAA GAG GTA TTG ACC A	
MdActin (MDP0000752428)-F	AAC AAT GCT AGG GAA CAC GGC TCT	
MdActin (MDP0000752428)-R	ACA GGA AGT AGA AGA TGG CGG ACA	
pCAMBIA2300-MdSPSB2-F	ATG GCG GGA AAC GAC TGG CTA	Vector construction
pCAMBIA2300-MdSPSB2-R	TTT TCG GTG GAC GAA ATG CAT ATA CC	
pCAMBIA2300-MdSPSC2-F	ATG AAG ACA CTA ATA CTT GGC AAC AGA	
pCAMBIA2300-MdSPSC2-R	AAA TCC CTT GAT CCC ACG TGC TT	
pTRV2-MdSPSB2-F	CTC GCC CGT AAA AAG AAG CAG AT	

pTRV2-MdSPSB2-R	CAT GCA CGC TGA TTA GGA CAA TG
pTRV2-MdSPSC2-F	CCC AGC TCT CGT GGA ACC T
pTRV2-MdSPSC2-R	GTG GAT GTT CTT TAG GCC GTT CT
